# Cross-species single-cell transcriptomic analysis of animal gastric antrum reveals intense porcine mucosal immunity

**DOI:** 10.1186/s13619-023-00171-w

**Published:** 2023-08-01

**Authors:** Xiaodan Wang, Fan Hong, Haonan Li, Yalong Wang, Mengxian Zhang, Shibo Lin, Hui Liang, Hongwen Zhou, Yuan Liu, Ye-Guang Chen

**Affiliations:** 1 The State Key Laboratory of Membrane Biology, Tsinghua-Peking Center for Life Sciences, School of Life Sciences, Tsinghua University, Beijing, 100084 China; 2 Guangzhou Laboratory, Guangzhou, 510005 China; 3 Guangzhou Institutes of Biomedicine and Health, Chinese Academy of Sciences, Guangzhou, 510530 China; 4 The First Affiliated Hospital of Nanjing Medical University, 300 Guangzhou Road, Nanjing, 210029 Jiangsu China; 5 School of Basic Medicine, Jiangxi Medical College, Nanchang University, Nanchang, 330031 China

**Keywords:** Single cell RNA-seq, Gastric antrum, Cross-species, Porcine immunity

## Abstract

As an important part of the stomach, gastric antrum secretes gastrin which can regulate acid secretion and gastric emptying. Although most cell types in the gastric antrum are identified, the comparison of cell composition and gene expression in the gastric antrum among different species are not explored. In this study, we collected antrum epithelial tissues from human, pig, rat and mouse for scRNA-seq and compared cell types and gene expression among species. In pig antral epithelium, we identified a novel cell cluster, which is marked by high expression of AQP5, F3, CLCA1 and RRAD. We also discovered that the porcine antral epithelium has stronger immune function than the other species. Further analysis revealed that this may be due to the insufficient function of porcine immune cells. Together, our results replenish the information of multiple species of gastric antral epithelium at the single cell level and provide resources for understanding the homeostasis maintenance and regeneration of gastric antrum epithelium.

**Supplementary Information:**

The online version contains supplementary material available at 10.1186/s13619-023-00171-w.

## Background

As an important digestive organ, the stomach secretes acids and enzymes, performs the function of food storage and digestion, and kills harmful microorganisms. Structurally, the human stomach can be divided into corpus and antrum, which show differences in epithelial cell type and function (Khurana and Mills 2010; Kim and Shivdasani 2016). The corpus can secrete acids (Castro et al. 2014), while antrum secretes mucus and certain hormones, especially gastrin. which stimulates corpus parietal cells to secrete gastric acid and facilitates the motility of the antrum and intestine (O'Connor and O'Morain 2014). Each antral gland unit consists of pit mucous cells, rare tuft cells, rare parietal cells, isthmus, endocrine cells and basal gland mucous cells (Willet and Mills 2016). The isthmus regions of the antrum contain proliferating cells and stem cells. Although the cell types in the gastric antrum are generally clear, the structure and size thereof in different species vary greatly due to the differences of their diet habits (Fothergill et al. 2019; Kararli 1995). At present, how different species differ in their cell composition and gene expression in the antrum remains obscure.

Different organisms, including mice, have been widely used as models to understand human disease development. However, there are limitations as the model organisms cannot fully mimic the human pathological process. For instance, mice infected with *Helicobacter pylori* only lead to mild gastritis and do not develop into ulcers or stomach cancer like humans (Ansari and Yamaoka 2022; Dey et al. 2021). The difference in gastric structure between mouse and human limits the use of mouse models for experimental *H. pylori* infection. Therefore, determining the differences in cell composition and gene expression among different species of gastric antrum is crucial for understanding gastric organ regeneration and homeostasis.

Most of cross-species cell type analyses were performed by antibody-based immunostaining, which are suffered from the limitation of the antibody efficiency in different species (Giladi and Amit 2018). The single-cell RNA sequencing (scRNA-seq) has solved this problem and been applied to cross-species comparison of multiple tissues (Elyada et al. 2019; Geirsdottir et al. 2019; Li et al. 2022). In this study, we collected antrum epithelial tissues from human, pig, rat and mouse for scRNA-seq. Our data provide a landscape of gastric antrum epithelial cells from the four species and reveal a new cluster of cells in pig antral epithelium. Gene expression analysis indicates that pig antral epithelium may have stronger immune function.

## Results

### ScRNA-seq analysis reveals the landscape of epithelial cells in the gastric antrum in human, pig, rat and mouse

**Fig. 1:**
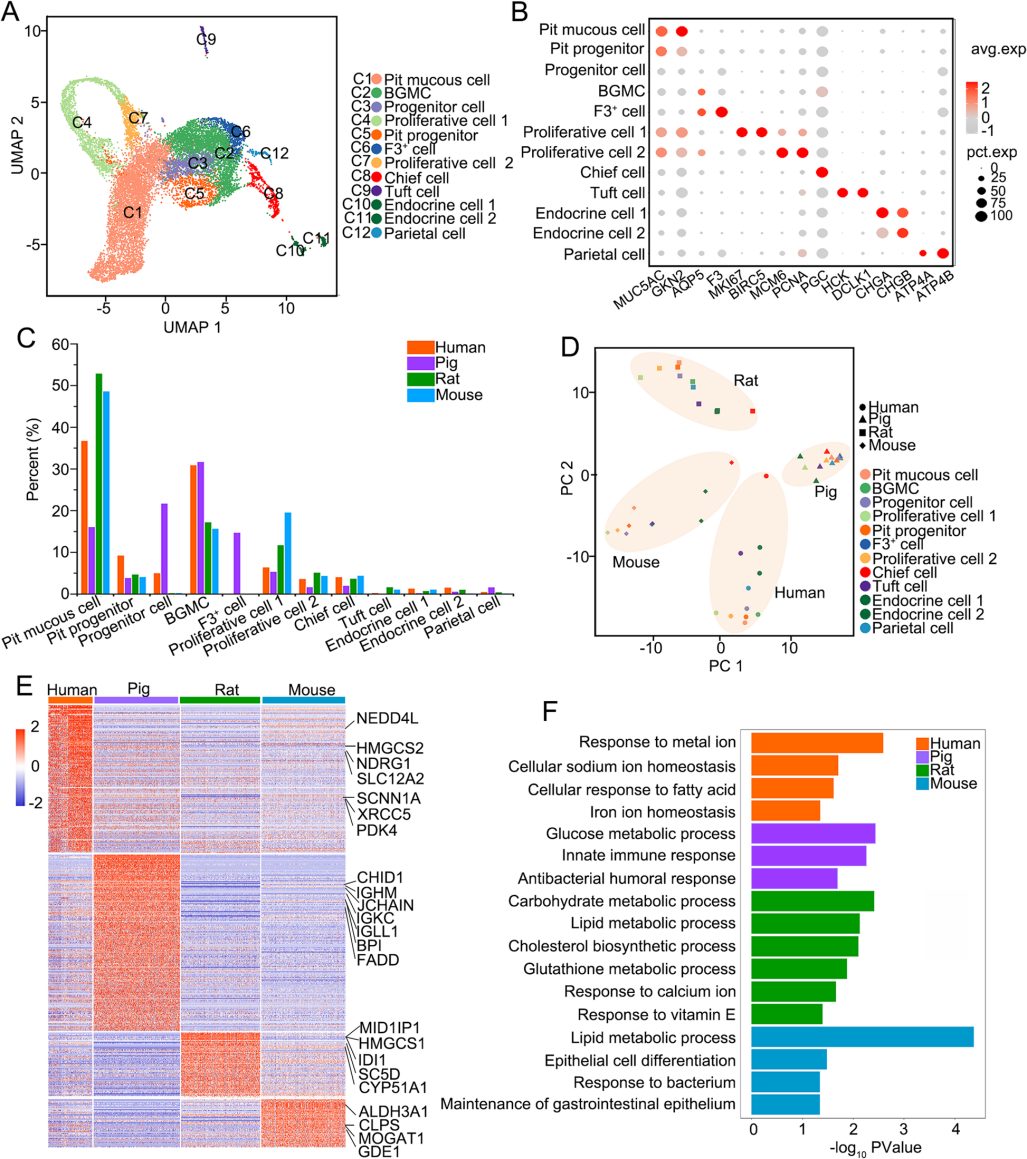
scRNA-seq analysis reveals the landscape of epithelial cells in gastric antrum in human, pig, rat, and mouse. **A** scRNA-seq analysis of gastric antral epithelium from human, pig, rat, and mouse as visualized by UMAP, all cells can be divided into 12 clusters. **B** Dot plot showing scaled expression level (color scale) and percent of expressing cells (point diameter) of marker genes in each cell cluster. **C** The cell ratio of each cell type in the gastric antral epithelium of the four species. **D** PCA plot of all cell types for each species. **E** Heatmap shows the differentially expressed genes in gastric antral epithelium of different species based on scRNA-seq. **F** Bar plot shows the functions of the highly expressed genes in the gastric antrum of each species

To compare the morphological structures of the antrum epithelial glands in human, pig, rat and mouse, we performed Hematoxylin and Eosin (H&E) staining, and found that the length of antrum glands in pig is similar to that in human (Fig. S1A). Moreover, immunofluorescence staining showed that the structure of pig and human glands exhibited similarities in distribution of pit cells, stem cells and other cell types. UEAI^+^ pit cells were enriched in the upper region of antral glands in human, pig and rat, but distributed along the gland in mouse (Fig. S1B and C). AQP5^+^ antrum stem cells were predominantly located at the base of the gastric glands in human, pig and mouse, but enriched in the upper region of rat antral glands. Conversely, HK-Atpase-β^+^ parietal cells were scarce in pig antral glands, but present in human and mouse antral glands. In order to gain a better understanding of antral epithelium functions in the 4 species, we performed scRNA-seq analysis as show in Figure S2. After quality control, 13,516 cells are used for subsequent analysis, the scRNA-seq library of each species has more than 2000 qualified cells, and the average number of genes exceeds 2500. The detailed cell number and gene number for each species were shown in Figure S3A-C and Table S1. Through unsupervised clustering analysis, all cells were divided into 12 clusters (Fig. 1A). According to the gene expression pattern and known markers (Bockerstett et al. 2020; Busslinger et al. 2021), we defined these 12 clusters as 9 cell types: pit mucous cell (*GKN1*, *MUC5AC*), pit progenitor cell, progenitor cell, basal gland mucous cell (*AQP5*), proliferative cell (*MKI67*, *BIRC5*, *MCM6*, *PCNA*), chief cell (*PGC*), tuft cell (*HCK*, *DCLK1*), endocrine cell (*CHGA*, *CHGB*) and parietal cell (*ATP4A*, *ATP4B*) (Figs. 1B and S3D). Furthermore, the proliferative cells could be divided into two subtypes: the proliferative cell 1 cluster showed high expression of *MKI67* and *BIRC5*, and the proliferative cell 2 cluster highly expressed cell cycle marker genes such as *MCM6*, *MCM5* (Fig. S3D). Two cell clusters highly expressed *AQP5*, which has been reported to be expressed in basal gland mucous cells (Tan et al. 2020). We found one of the them also highly express gene *F3* (Fig. 1B) and thus temporarily named it as F3^+^ cells, and the other cluster was named as basal gland mucous cells (BGMCs). BGMCs were uniformly distributed among four species, but F3^+^ cells were unique to pig (Fig. 1C; Table S2). Progenitor cells with low expression of *F3* and *CLCA1* only existed in the gastric antral epithelium of human and pig. Furthermore, we found that the proportions of pit mucous cells and proliferative cells are higher in rat and mouse, while human and pig have higher proportion of BGMCs. These results together indicate that human antrum epithelial cells were more similar to those of pig in cell composition, while antrum epithelial cells of rat and mouse were close to each other.

To better analyze the gastric antrum of the four species, we performed PCA analysis and found that the intra-species differences of pig gastric antral epithelial cells were minimal among the four species (Fig. 1D). In the remaining three species, the chief cells and EECs were quite different from pit mucous cells and TA cells. Chief cells, which were a class of cells that secreted proteases in the gastric epithelium, exhibited the least difference among species. This suggests that in different species, the function of proteases secreted by chief cells of gastric antrum may be conserved. PCA analysis also revealed that the gastric antral epithelium of pig and mouse was more similar to human.

To explore the functional differences in gastric antral epithelium among different species, we performed functional enrichment analysis of the differentially expressed genes among species. The results showed that genes related to metal ion homeostasis, such as *NEDD4L* (Anta et al. 2016; Kang et al. 2015) and *SCNN1A* (Hummler and Beermann 2000; Ludwig et al. 1998), were highly expressed in human gastric antral epithelium, while many immune-related genes, such as *CHID1* (Huang et al. 2011), *IGHM* (McHeyzer-Williams et al. 2011; Schroeder and Cavacini 2010), *IGKC* (McHeyzer-Williams et al. 2011; Schroeder and Cavacini 2010), *IGLL1* (McHeyzer-Williams et al. 2011; Schroeder and Cavacini 2010), *BPI* (Elsbach 1998) and *FADD* (Bolze et al. 2010), were specifically and highly expressed in pig (Fig. 1E and F). The genes related to lipid metabolism were detected in rat and mouse epithelium, such as *MID1IP1* (Ding et al. 2022; Gaudet et al. 2011), *HMGCS1* (Rokosz et al. 1994), *SC5D* (Matsushima et al. 1996) and *IDI1* (Gaudet et al. 2011) in rat, and *ALDH3A1* (Lee et al. 2019), *CLPS* (Davis et al. 1991), *MOGAT1* (Gaudet et al. 2011) and *GDE1* (Zheng et al. 2000) in mouse (Fig. 1E and F). The difference in gene expression of the gastric antrum epithelium across species may be due to gastric antrum epithelium function caused by diet habits.

### Identification of a species-specific cell cluster in pig antral epithelium

**Fig. 2:**
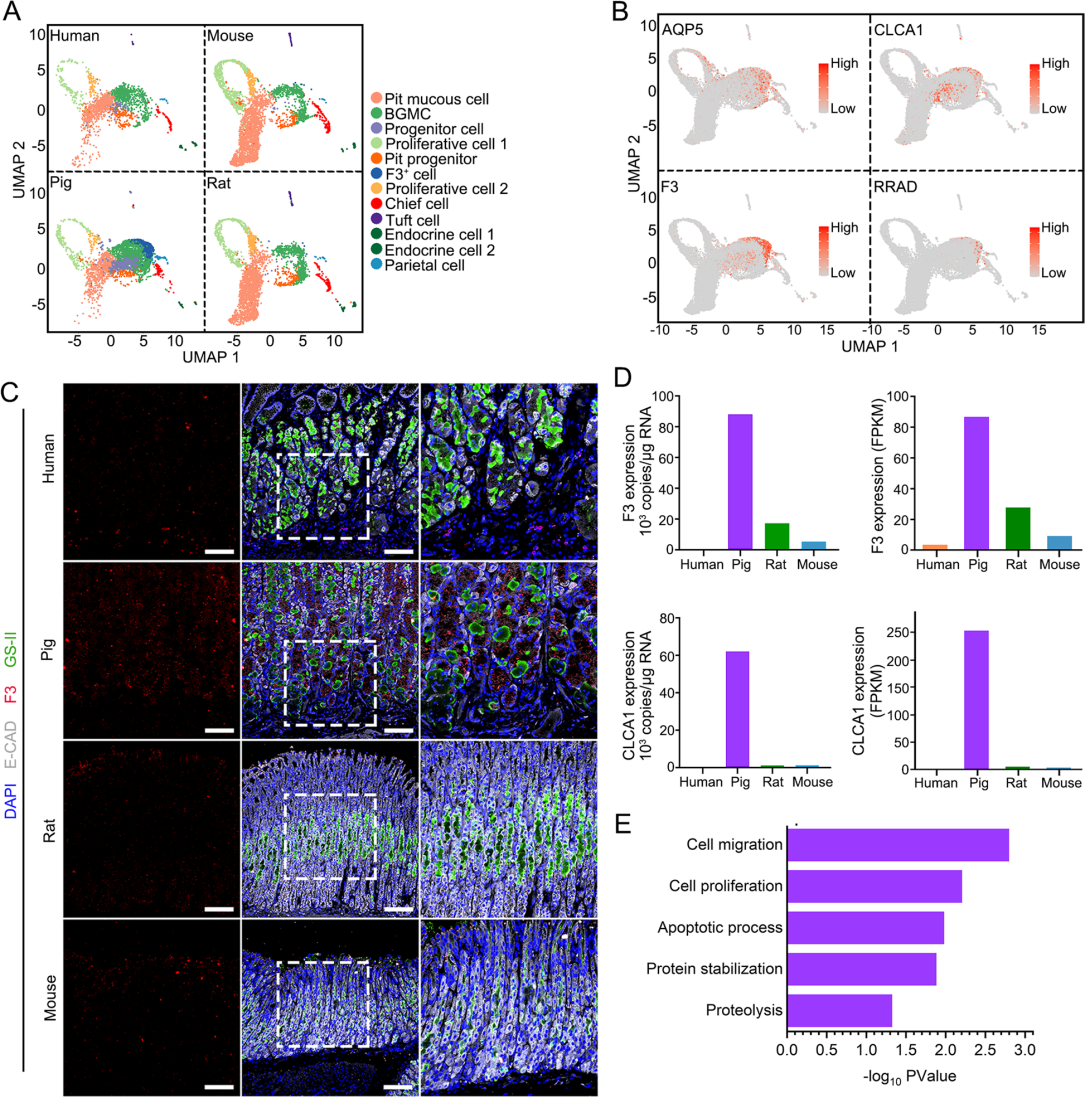
Identification of a species-specific cell cluster in pig antral epithelium. **A** Dim plots show the gastric antrum epithelial cells from human, pig, rat, and mouse, respectively. **B** Dim plots show the expression of F3^+^ cell marker genes, AQP5, CLCA1, F3 and RRAD. **C** Expression pattern of F3, GS-II and E-cadherin in the antrum of human, pig, rat, and mouse, indicated by immunofluorescence staining (*n* = 3). Scale bars, 100 μm. **D** The copy number in 1 μg RNA and the expression of F3 and CLCA1 (*n* = 3 independent experiments) (left). The FPKM of F3 and CLCA1 in antrum organoids (right). **E** Function enrichment analysis of the highly expressed genes in F3^+^ cells identified by scRNA-seq

We found that a cell cluster, named F3^+^ cells, was exclusively existed in pig gastric antral epithelium (Figs. 1C and 2A). F3^+^ cells were found to be distributed from the neck to the bottom of gastric glands in the pig antrum (Fig. S4A). The genes *F3*, *CLCA1* and *RRAD* were specifically and highly expressed in this cell cluster (Fig. 2B). These cells appeared to be different from the known basal gland mucus cells that we named BGMCs, which were distinct from F3^+^ cells as GS-II labeling BGMCs was rarely co-localized with F3 (Fig. S4B). F3^+^ cells and BGMCs displayed very different gene expression (Fig. S4C). Immunostaining confirmed the presence of *F3*-positive cells in pig gastric antral epithelium but not in human, rat and mouse (Fig. 2C). Furthermore, we examined the expression of *F3* and *CLCA1* by qPCR and bulk RNA-seq in organoids derived from gastric antral epithelium of the four species and observed that both of these two genes were highly expressed in the pig antral epithelium (Fig. 2D).

Gene ontology (GO) enrichment analysis revealed that genes specifically and highly expressed in F3^+^ cells were mainly involved in cell migration, cell proliferation, protein stabilization and proteolysis (Fig. 2E). It is worth mentioning that previous studies on submucosal glands of pig lung showed that the serous cells also highly expressed genes such as *AQP5*, *F3*, *ATF3* and *PIGR*, which were very similar to F3^+^ cells (Yu et al. 2022). As serous cells are mainly involved in digestion and defense (Basbaum et al. 1990), we speculate that F3^+^ cells might perform similar functions in pig gastric antrum and be a unique component in the pig gastric antrum epithelium that is involved in food digestion and protects tissues from external invasion.

### The antrum epithelial cells of pig express immune genes

**Fig. 3:**
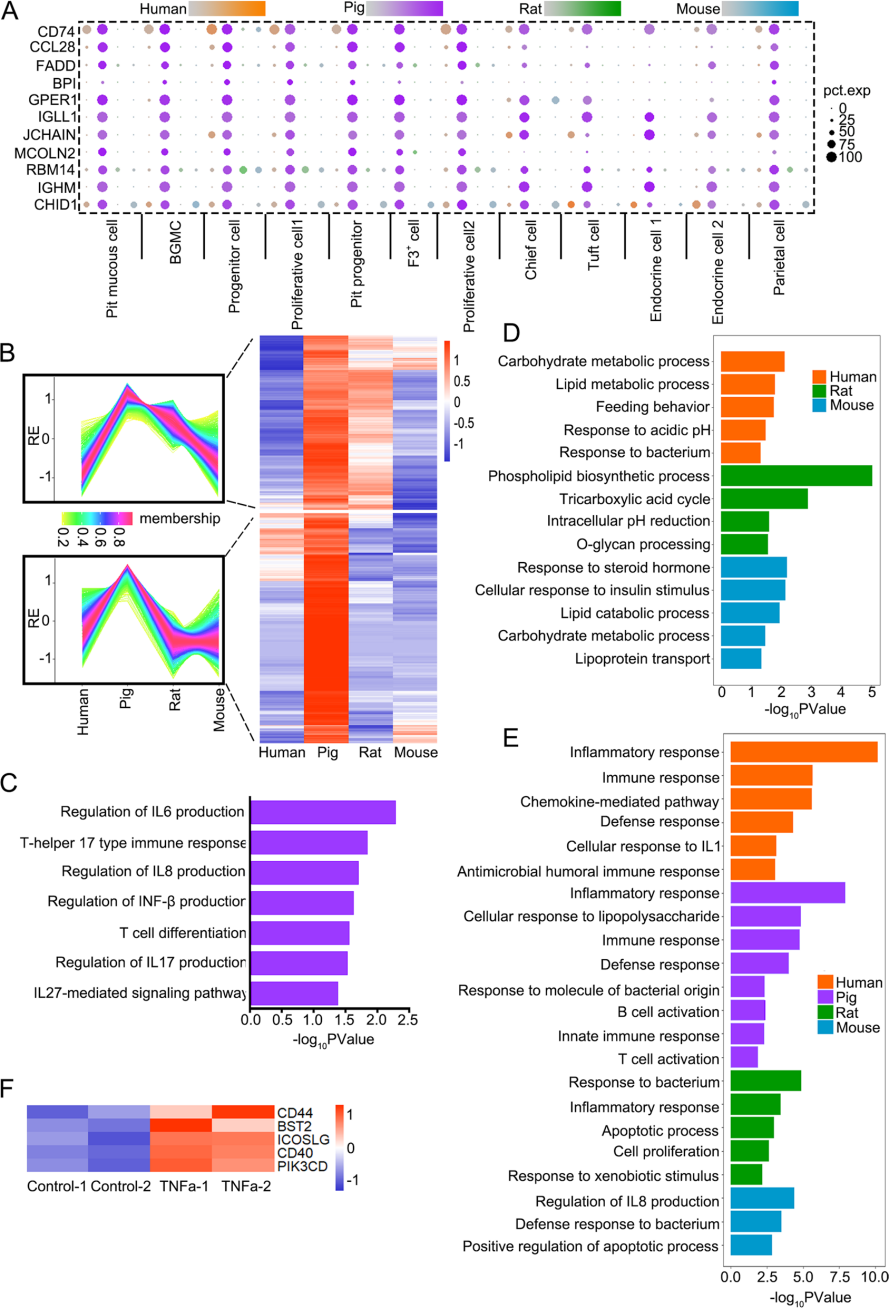
The pig antrum epithelial cells highly express immune response genes. **A** Dot plot shows the expression level (color scale) and expressing cells (point diameter) of immune response genes in four species revealed by scRNA-seq. **B** Bulk RNA-seq analysis indicates that the highly expressed genes in pig gastric antral organoids can be divided into 2 modules according to their expression dynamics. The change trend in each module is shown in the line chart on the left. **C** Function enrichment analysis of genes highly expressed in pig gastric antral organoids identified by bulk RNA-seq. **D** Function enrichment analysis of genes highly expressed in gastric antral organoids of human, rat and mouse identified by bulk RNA-seq. **E** Functional enrichment analysis of the up regulated genes in the gastric antral organoids treated with TNFα. **F** Expression heatmap of genes related with activation of T cells and B cells in control and TNFα treated organoids based on bulk RNA-seq

By analyzing genes differentially expressed in human, pig, rat and mouse through scRNA-seq, we observed that highly expressed genes in human gastric antrum epithelium were related to metal ion homeostasis, and genes highly expressed in rat and mouse were related to lipid metabolism (Fig. 1F). Many immune genes such as *CD74* (Gaudet et al. 2011), *BPI*, *GPER1* (Notas et al. 2020), *IGHM* and *JCHAIN* were highly expressed in pig antral epithelium. Immunostaining confirmed that CD74 was expressed in the epithelial cells of both human and pig but not in rat and mouse, and the expression is higher in pig antrum epithelial cells than that in human (Fig. S5A). Bulk RNA-seq of antrum epithelial organoids further validated higher expression of most of these genes in pig scRNA-seq library (Fig. S5B). In addition, we found that many other immune function genes were highly expressed in pig organoids, such as *CD79A* (Gaudet et al. 2011), *CD40* (Gaudet et al. 2011) and *IL17D* (Starnes et al. 2002) (Figs. 3A, B and S5C; Table S3). GO enrichment analysis revealed that genes highly expressed in pig were involved in the production of interleukin (IL) genes, INF-β and immune response (Fig. 3C). In the other three species, however, highly expressed genes were mainly involved in nutrient metabolism and pH regulation, except for the antibacterial-related genes in human (Figs. 3D and S5D). These results indicate that under normal physiological conditions, pig antrum epithelial cells have strong immune capacity, which may be related to their complex eating habits and living environment.

To test the immune function of gastric antrum epithelium from different species, we treated organoids from different species with TNFα and detected the differentially expressed genes. GO enrichment analysis showed that TNFα upregulated the expression of immune response genes in all species (Fig. 3E). In particular, we observed enhanced expression in pig of the genes related to the activation of T cells and B cells, such as *ICOSLG* (Khayyamian et al. 2002) and *PIK3CD* (Fung-Leung 2011) (Fig. 3F; Table S4). Interestingly, the up-regulated genes induced by TNFα in four species were distinct, and more genes were upregulated in human and pig (Fig. S5E), suggesting that the antral epithelium among different species may have distinct immune response, and stronger immune response may take place in human and pig.

### Immune cells in the pig antrum are highly proliferative with low immunity

**Fig. 4:**
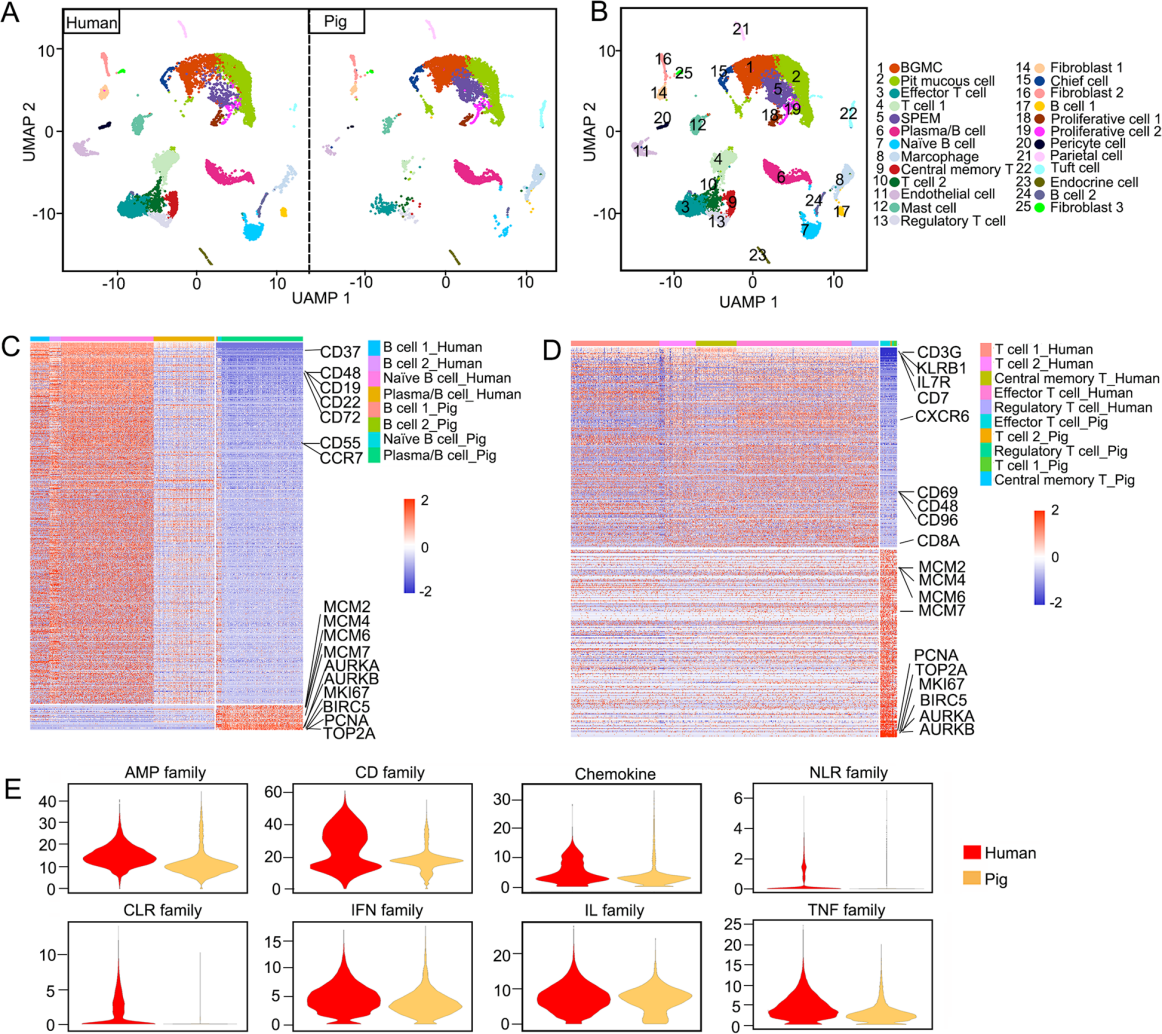
T cells and B cells in pig antrum show high proliferation and low immunity compared to those in human antrum. **A**, **B** scRNA-seq analysis of stroma and epithelium from human and pig as visualized by UMAP. **C**, **D** Heatmap shows the differentially expressed genes of B cells (**C**) and T cells (**D**) between human and pig. **E** Violin plots show the overall expression of different immune family genes

The high expression of immune-related genes in the pig antral epithelium but not in human may be due to the difference in immune cells between human and pig. To examine this possibility, we extracted the cells from the stroma of human and pig gastric antrum for scRNA-seq (11,975 cells from human and 7199 cells from pig) (Fig. 4A). The cells were divided into 25 clusters (Fig. 4B), based on the expression of reported marker genes (Fig. S6A). Counting of immune cells (including B cells, T cells, macrophages and plasma cells) revealed that the proportion of B cells and T cells in pig was lower than that in human (Fig. S6B; Table S5).

Analysis of differentially expressed genes in B cells and T cells uncovered that the genes highly expressed in human were mainly related to the activation and function of B cells and T cells, whereas genes highly expressed in pig were mainly involved in cell proliferation (Fig. S6C-E). For instance, the proliferation genes *MKI67*, *BIRC5*, *TOP2A* and *PCNA* were highly expressed in pig T cells and B cells, while the immune-related genes *IL7R*, *CD7* and *CXCR6* high in human (Fig. 4C and D). Indeed, *MKI67* and *PCNA* signals were detected in epithelial and non-epithelial cells in human and pig antrum glands (Fig. S7A and B), and in non-epithelial cells, *MKI67* and *PCNA* were higher in pig than in human (Fig. S7C). To better compare the immune competence of the immune cells in the two species, we calculated the expression of different immune family genes in T and B cells. The results indicated that the overall expression of all the immune family genes was higher in human than that in pig (Fig. 4E; Table S6). Taken together, these analyses suggest that the immune cells in the pig gastric antrum are highly proliferative and are maintained in a small proportion with insufficient immune function.

### Signaling interaction between epithelial and stromal cells in pig and human gastric antrum

**Fig. 5:**
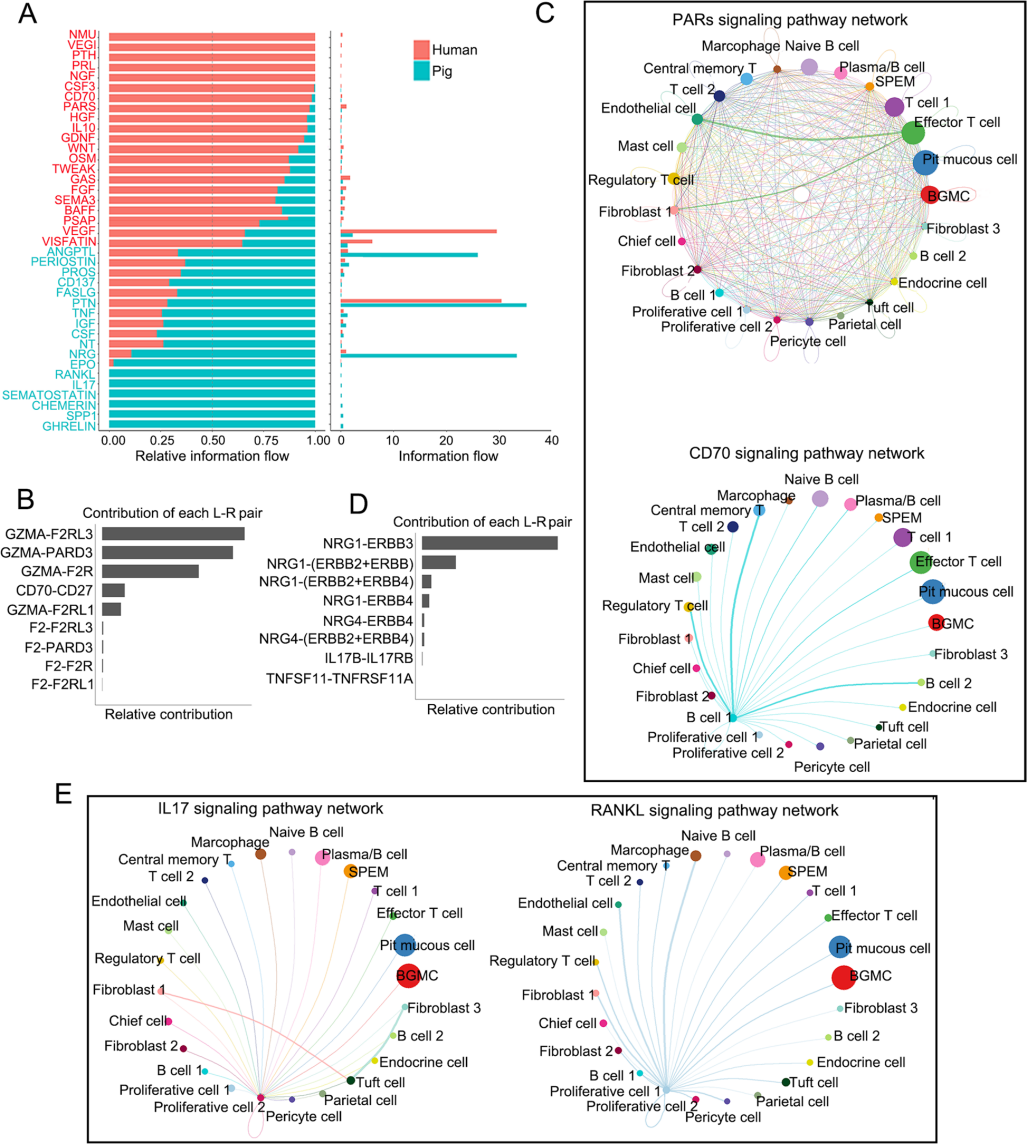
Comparison of signaling networks between epithelial and stromal cells in pig and human gastric antrum. **A** Relative strength of different signaling pathways in human and pig gastric antrum. **B** Ligand-receptor pairs have higher expression or specifically present in human gastric antrum. **C** Circle plots show PARs and CD70 signaling pathways in human gastric antrum. **D** Ligand-receptor pairs have higher expression or specifically present in pig gastric antrum. **E** Circle plots show IL17 and RANKL signaling pathways in human gastric antrum

The difference in immune function execution between human and pig antrum could be the result of different cellular signaling regulation. We analyzed the interaction of epithelial cells and stromal cells in human and pig, and found that many signaling pathways were different between the two species. The ligand-receptor pairs highly expressed or specifically present in the human gastric antrum were related to the generation, maturation, maintenance and function of immune cells, while enriched pairs in pig were mainly related to the growth and differentiation of epithelial cells, as well as the anti-inflammatory and antibacterial, proliferation of immune cells (Fig. 5A). For instance, PAR and CD70 signaling components were enriched in human, while IL17 and RANKL in pig. The ligand-receptor pairs GZMA-F2RL3, GZMA-PARD3 and GZMA-F2R belong to PAR signaling, and CD70-CD27 belongs to CD70 signaling (Fig. 5B and C). GZMA is a T cell- and natural killer cell-specific serine protease that may function as a component necessary for the lysis of target cells by cytotoxic T lymphocytes and natural killer cells (Gershenfeld et al. 1988). The CD70 signaling was mainly involved in the interaction between T cells and B cells (Fig. 5C). Upon CD27 binding, CD70 induces the proliferation of co-stimulated T-cells and enhances the generation of cytolytic T-cells (Flieswasser et al. 2022). In the human gastric antrum, the ligands of PAR signaling mainly existed in effector T cells, and the ligand-receptor pairs promoted the maturation of T cells and B cells, which is consistent with the strong immune competence in human gastric antrum.

Most of signaling molecules with high expression in pig were mainly related to the differentiation and maturation of epithelial cells. The NRG1-ERBB3 pair was highly expressed in pig antrum (Fig. 5D), and NRG1 might promote growth and differentiation of epithelial cells (Ieguchi et al. 2010). IL17 family is primarily involved in host defense against extracellular bacteria and fungi by inducing neutrophilic inflammation (McGeachy et al. 2019). TNFSF11-TNFRSF11A, belonging to RANKL signaling, augments the ability of dendritic cells to stimulate naive T-cell proliferation and may regulate interactions between T-cells and dendritic cells (Wong et al. 1997) (Fig. 5D and E). In the pig gastric antrum, RANKL signals mainly came from proliferative epithelial cells and might promote the proliferation of receptor cells, including T cells and B cells (Fig. 5E). Consistent with the gene expression profiling, the ligand-receptor pairs in the human gastric antrum are involved in the maturation of T cells and B cells and maintain their immune function, while the ligand-receptor pairs in the porcine gastric antrum mainly promote the proliferation of T cells and B cells, but not the immune function.

## Discussion

In this study, we investigated the transcriptome of the gastric antrum epithelial tissues from human, pig, rat and mouse and displayed the landscape of gastric antral epithelium at the single cell level, with cross-species comparison on the cell type and gene expression. Previous studies have revealed great differences in the structure of stomach caused by different diet habits among species. Compared to rodents, humans do not have the forestomach with a squamous epithelium, only possessing the glandular stomach with a simple columnar epithelium (Kim and Shivdasani 2016). The gastric antrum and corpus are the main functional area of the human stomach, and the main cell types in the gastric antrum epithelium have been described (Karam and Leblond 1993; Willet and Mills 2016). In our study, we have identified a new cluster of cells (F3^+^ cells) in pig gastric antral epithelium, which highly expressed *AQP5*, *F3*, *CLCA1* and *RRAD*. Due to the limitation of scRNA-seq depth, further functional analysis of this cell cluster was impossible. We have also tried to investigate the potential function of F3^+^ cells in an organoid system. However, pig antral organoids predominantly consist of proliferative cells, and F3 expression was not detected due to the specific culture conditions requiring high levels of Wnt3a. As F3^+^ cells express the markers of serous cells in submucosal glands of pig lung (Yu et al. 2022), we speculate that F3^+^ cells might perform similar functions in pig gastric antrum by promoting digestion and resisting external invasion.

Pig is regarded to be a good model for studying human gastric ulcer (Fuller and Boenker 1968). *H.pylori* originates from non-human primates and can infect humans, it is a common pathogen in zoonosis (Flahou et al. 2018; Smet and Menard 2020). Therefore, it is interesting to compare the immune function in the gastric antrum between human and pig. Our study suggests that pig antral epithelium may bear stronger immune function, while porcine immune cells possess insufficient immune capacity. Despite that F3^+^ cells are not present in pig antrum organoids, other epithelial cells in pigs exhibit higher expression levels of immune response genes compared to other species, suggesting that organoids are still a valuable system for studying the function of gastric epithelium. The analysis of signaling networks between epithelium and stroma shows that the signaling networks in human can promote the generation, maturation and maintenance of the immune cell functions, while signaling networks in pig are mainly related to the proliferation of immune cells. We speculate that strong immune function in pig gastric antrum confers epithelial cells the resistance to external invasion due to the complexity of diet habits and living environment.

## Methods

### Human gastric tissue collection and ethics statement

Human antrum tissue adjacent with pyloric about 2–3 cm from patients who under gastric sleeve resections were obtained from Department of General Surgery, The First Affiliated Hospital of Nanjing Medical University, (Nanjing, China) with prior approval of the ethics committee of The First Affiliated Hospital of Nanjing Medical University, Nanjing (2017-SR-171.A2). Anonymized samples (Table S2) were obtained from a total of 4 patients with body mass index > 31 kg/m^2^.

### Animals

All mice and rats used in this study were maintained in the Animal Facility of Tsinghua University. The experiment protocols were approved by IACUC (Institutional Animal Care and Use Committee) of Tsinghua University (19-YGC). Bama miniature pigs were used in this study from Beijing Farm Animal Research Center (affiliated to Institute of Zoology, Chinese Academy of Sciences). The experimental protocols were approved by the Animal Ethics Committee of the Institute of Zoology, Chinese Academy of Sciences (IOZ20180061).

### Antrum samples preparation for scRNA‑seq

The antrum tissue was extracted from four adult human, three adult mice (10 weeks), three adult rats (3 months old) and three adult pigs (6 months old). Fragments of the gastric antrum region were isolated from different species. The digestion protocol of the gastric epithelial cells was varied in species. The antrum fragments of human, mouse, rat and pig antrum was incubated with 20 mM EDTA digestion for 25 min at 4 °C. Then, the gastric glands from these species were scraped from tissue and collected. Then for human, mouse and rat antrum were followed by Tryple (Invitrogen) digestion for 10–15 min at 37 °C. For pig antrum, the epithelial cells were incubated with 10,000 U/mL collagenase IV (Thermo Fisher), 30 mg/ml dispase II (Roche), and 1 M hepes (MACKLIN), 2500 U/ml DNase I (KeyGEN) in Advanced DMEM F12 for 15 min at 37 °C. After filtering with 40 μm strainer and stained with propidium iodide (PI; 5 μg/ml), PI-negative cell cells were sorted by FACS (Beckman). Single cells were loaded onto the single cell chip from 10X genomics Chromium Single Cell 3′ Solution. The cDNA library was constructed according to instruction and sequenced by Illumina Novaseq 6000 sequencer (Illumina, San Diego, CA, USA) with paired-end 150-bp reads.

### Antrum organoid culture from human, mouse, rat and pig

The antrum tissue was cut from stomach of these species and washed by cold PBS for 5–6 times to remove the contaminant. After incubation with 20 mM EDTA for 25 min at 4 °C, gastric glands were carefully scraped and collected. Then, after centrifugation (3 min at 1000 rpm), the gastric glands were embedded into Matrigel (BD Biosciences) and seeded on 24-well plate. After polymerization, the culture medium was added. Advanced DMEM/F12 was supplemented with 2 mM GlutaMAX, 1 mM N-acetylcysteine, 1X N2, 1X B-27 and penicillin/streptomycin to prepare a basal medium (all from Thermo Fisher). The organoid culture medium for human and mouse antrum included 50 ng/mL EGF (Peprotech), 100 ng/mL Noggin (Novoprotein), 500 ng/mL R-spondin-1 (Novoprotein), 5 μM CHIR-99021 (Selleck), 10 nM Gastrin (TOCRIS), 100 ng/mL FGF10 (Novoprotein), 10 μM SB431542 (Selleck) and 10 μM Y-27632 (Selleck) in basal medium. The organoid medium for rat and pig antrum was supplemented with 30% wnt3a conditional medium. Growth medium was replaced every 3–4 days.

### Stimulation experiments

For TNFα stimulation, organoids derived from human, mouse, rat and pig antrum were prepared and cultured in 24-well plate. Then, after 2 days treatment of 10 ng/mL TNFα, organoids were removed from Matrigel and lysed for RNA isolation.

### Hematoxylin‑eosin (H&E) staining

The gastric tissue was fixed using 4% formalin overnight and subsequently embedded in paraffin. To prepare the sections for analysis, 5 μm thick sections were deparaffinized using a series of graded alcohols and isopropanol. The sections were then subjected to H&E staining using a commercial H&E staining kit (Beyotime). Briefly, the sections were stained with hematoxylin solution for 6 min and followed by treatment with 1% acid alcohol for 2 s. Subsequently, the sections were stained with eosin for 2 min using the eosin solution provided in the H&E staining kit (C0105, Beyotime).

### Immunofluorescence

Immunofluorescence was performed as previously described (Qi et al. 2017). Briefly, the antrum tissues from human, mouse, rat and pig were washed in cold PBS and were fixed in 4% paraformaldehyde for overnight at room temperature. Then paraffin-embedded antrum sections were de-paraffinized in xylene and dehydrated by a graded alcohol series, followed by antigen retrieval. Next, after washing by PBS for 3 times, the sections were blocked and permeabilized by 0.3% Triton X-100 and 5% BSA for 1 h at room temperature. Then, the sections were incubated with primary antibodies overnight at 4 °C. The fluorescein-labeled secondary antibodies (Life Technologies, 1:300) and 4′, 6-diamidino-2-phenylindole (DAPI) were added for 1 h at room temperature next day. The images were acquired from Olympus FV3000 Laser Scanning Microscope.

### Antibodies

Rabbit anti-Tissue Factor antibody (1:200, ab228968; Abcam), rabbit anti-AQP5 antibody (1:200, ab92320; Abcam), rabbit anti-Ki67 (1:200, ab15580; Abcam), mouse anti-PCNA (1: 300, sc-56; Santa Cruz), mouse anti-HK-Atpase-β (1:300, sc-374094; Santa Cruz), rabbit anti-E-cadherin (1: 300, 3195; Cell Signaling), rabbit anti-CD74 (1:300, ab64772; Abcam) and mouse anti-E cadherin (1:200, 610182; BD Biosciences). FITC-conjugated UEAI lectin (1:2000, L32476; Thermo Fisher) and Lectin GS-II conjugate with Alexa Fluor 647 (100 μg/mL, L32451; Thermo Fisher).

### RNA extraction and quantitative RT–PCR

The total RNA from organoids was extracted by RNeasy Mini Kit (Qiagen). The cDNA was obtained by 1 st Strand cDNA Synthesis Super Mix (Novoprotein). Then, real-time PCR reactions were performed using qPCR SuperMix Plus (Novoprotein) in triplicates on a LightCycler 480 (Roche). The primers of selected gene were shown in Table S3. The experiments were performed with three biological replicates.

### Bulk RNA-seq

After total RNA extraction and then subjected to high-throughput sequencing on an Illumina Novaseq PE150 platform. RNA-seq was carried out with two biological replicates.

### Ortholog gene selection

To compare transcription between species, we first created a gene ortholog list using the human genes as the reference. We download homologous gene lists from Ensemble BioMart (https://asia.ensembl.org/biomart/martview/efb2456d7ea6a4d37b6a2a9f03499a88). Other three species were compared to human and a high-quality ortholog genes list was extracted. To account for gene paralogs and gene-duplication events, an aggregated table of “meta-genes” was created. Each meta-gene may include all gene symbols homologous to one human gene. For each organism, read counts were combined across all manifestations of each meta-gene. Finally, we sorted out 14,331 orthologous genes across 4 species, including 13,343 “1–1-1–1” orthologous genes and 988 “1-many” genes.

### scRNA-seq low-level processing and filtering

Raw reads were aligned to the different species genome (Human: GRCh38/hg38, Pig: Sscrofa11.1, Rat: Rnor_6.0, Mouse: GRCm38/mm10), and Cell Ranger (v3.1.0) (Zheng et al. 2017) was used to estimate unique molecular identifiers (UMIs). Raw aligned features were loaded and processed using the Seurat package (v4.0.2) (Hao et al. 2021) in R version 4.0.5. Low-quality cells were filtered if they expressed no more than 200 genes or with more than 20% of mitochondrial genes.

### scRNA-seq normalization and clustering

Data normalization was performed using Seurat “NormalizeData” and using “LogNormalize” as the normalization method (sacle.factor = 100000). Variable genes were detected using “FindVariableFeatures”. We used “FindIntegrationAnchors” to combine the scRNA-seq libraries of the four species. The scaled gene expression data were projected onto principal components (PCs). The first 30 PCs were used for non-linear dimensionality reduction using Uniform Manifold Approximation and Projection (UMAP). Clustering was performed using the “FindNeighbors” followed by the “FindClusters” functions. Marker genes for each cluster have been identified using “FindAllMarkers” function.

The four batches of scRNA-seq data from human, pig, rat and mouse were subjected to batch correction as described previously (Mayer et al. 2018). We use the canonical correlation analysis (CCA) strategy to find linear combinations of features across datasets that are maximally correlated. The shared correlation structure conserved among the four datasets. Based on the shared structure, all four batches of data were finally pooled into a single object for downstream analyses (Butler et al. 2018).

### scRNA-seq differential gene expression analysis

To identify signature genes of each cell types, functions “FindAllMarkers” and “FindMarkers” in Seurat were used. The function “FindMarkers” was used for identification of signature genes by comparing the cell type of interest to another specific group of cells. Functional enrichment analysis was performed using the online software DAVID (https://david.ncifcrf.gov) tool with default parameters. PCA analysis were performed using R software.

### RNA-seq analysis

RNA-seq reads were aligned to the human and mouse genomes available in Ensembl (release 95) using HISAT2 (v2.1.0) (Kim et al. 2019). The resulting SAM files were sorted and converted to BAM files using SAMtools (v1.9) (Li et al. 2009), and then passed to StringTie (v1.3.3b) (Frazee et al. 2015) for transcript assembly. Transcript expression levels (counts) were calculated and quantitative gene expression (FPKM) was obtained using Ballgown (v2.16.0) (Frazee et al. 2015; Pertea et al. 2016), and genes with FPKM less than 1 were discarded as unexpressed. Genes with |log2 FC| greater than 1 and *P* < 0.05 were considered differentially expressed. Functional enrichment analysis was performed using the DAVID online software tool (https://david.ncifcrf.gov) with default parameters.

## Supplementary Information

**Additional file 1: Fig. S1.** Structure of the antrum of human, pig, rat, and mouse. A H&E staining illustrating the structure of the antrum. Scale bar: 200 μm. B, C Expression of UEAI labeling pit cells, HK-Atpase-β labeling parietal cells (B), and AQP5 labeling antrum stem cells (C) in the antrum. Scale bar: 100 μm. **Fig. S2.** A graphical abstract showing the main workflow of this study. Schematic of stomach anatomy and cross-species scRNA-seq analysis in human, pig, rat and mouse. Dashed box indicates the gastric antrum epithelial region selected for scRNA-seq in all species. **Fig. S3.** scRNA-seq analysis reveals the landscape of epithelial cells in gastric antrum in human, pig, rat, and mouse. A The quality information of scRNA-seq from each species. B scRNA-seq analysis of gastric antral epithelium of human, pig, rat, and mouse as visualized by UMAP. C Violin plots show the range of genes identified by scRNA-seq in each species. D Expression levels of marker genes in each cell type. Pit mucous cell (GKN1, MUC5AC), basal gland mucous cell (AQP5), proliferative cell (MKI67, BIRC5, MCM6), chief cell (PGC), tuft cell (HCK, DCLK1), endocrine cell (CHGA, CHGB) and parietal cell (ATP4A, ATP4B). Color from gray to red indicates relative expression levels from low to high. **Fig. S4.** Distribution of F3^+^ cells in the pig antrum. A F3^+^ cells in the pig antrum glands, extending from the top to the bottom. B Expression of F3 and GSII in the pig antrum glands. Scale bar: 100 μm. C Heatmap of differentially expressed genes between BGMCs and F3^+^ cells. **Fig. S5.** The antrum epithelial cells of pig express high levels of immune response genes. A Expression of CD74 in the gastric antrum epithelial tissues of human, pig, rat, and mouse. B Heatmap shows the expression level of immune response genes in gastric antrum epithelial organoids of four species through bulk RNA-seq. C Heatmap shows the expression of immune function genes in gastric antrum epithelial organoids. D Heatmap shows the highly expressed genes in organoids of human, pig, rat, and mouse (left). The change trend in each module is shown in the line chart on the right. E Venn diagram indicates the up-regulated gene number of organoids treated with TNFα in four species. **Fig. S6.** T cells and B cells in pig antrum show high proliferation and low immunity compared to those in human antrum. A Dot plot showing scaled expression level (color scale) and percent of expressing cells (point diameter) of marker genes in each cell cluster. B The cell ratio of immune cells (including B cells, T cells, macrophages and plasma cells) in human and pig, based on scRNA-seq. C Dot plot showing scaled expression level (color scale) and percent of 8 expressing cells (point diameter) of immunity genes (left) and proliferating genes (right) in each cell cluster of human and pig. D, E Function enrichment analysis of highly expressed genes in B cells (D) and T cells (E) of human and pig. **Fig. S7.** Proliferative signal in antrum glands of human and pig A Expression of MKI67 in the antrum of human and pig. B Expression of PCNA in the antrum of both human and pig. C Quantification of MKI67^+^ and PCNA^+^ cells in nonepithelial cells per view in panels (A) and (B). Scale bar: 100 μm

**Additional file 2: Supplemental Table S1.** Cell number and gene counts of four species scRNA-seq libraries in Fig. S3A and B.

**Additional file 3: Supplemental Table S2.** The cell ratio of each cell type in the gastric antral epithelium of the four species in Fig. 1C.

**Additional file 4: Supplemental Table S3.** Bulk RNA-seq shows the FPKM of genes that have higher expression in pig gastric antrum in Fig. 3B.

**Additional file 5: Supplemental Table S4.** FPKM of genes related with activation of T cells and B cells in pig organoids of control and TNFα treatment in Fig. 3F.

**Additional file 6: Supplemental Table S5.** The cell ratio of each cell type in human and pig in Fig. S6B.

**Additional file 7: Supplemental Table S6.** The list of each immune family in Fig. 4E.

**Additional file 8: Supplemental Table S7.** Shows information about the human antrum samples.

**Additional file 9: Supplemental Table S8.** Shows quantitative PCR primers.

## Data Availability

The RNA-seq data and scRNA-seq data generated in this study are publicly available through the Gene Expression Omnibus (GEO) with the accession code GSE225276. All other data are available from the corresponding author on request. All codes that enable the main steps of the analysis are available from the corresponding author under request.

## Abbreviations

AQP5: Aquaporin 5
BGMC: Basal gland mucous cell
CCA: Canonical Correlation Analysis
CLCA1: Chloride channel accessory 1
F3: Coagulation factor III
PCA: Principal Component Analysis
RNA-seq: RNA sequencing
RRAD: Ras related glycolysis inhibitor and calcium channel regulator
scRNA-seq: Single cell RNA sequencing
TNFα: Tumor necrosis factor-α
UMAP: Uniform Manifold Approximation and Projection
