# Supplementary material for: Cross-species single-cell transcriptomic analysis of animal gastric antrum reveals intense porcine mucosal immunity
Source: Cell Regen. 2023 Aug 1;12:27. doi: 10.1186/s13619-023-00171-w (PMC10390400; doi:10.1186/s13619-023-00171-w)
Supplement: Supplementary file 1 — Additional file 1: Fig. S1. Structure of the antrum of human, pig, rat, and mouse. A H&E staining illustrating the structure of the antrum. Scale bar: 200 μm. B, C Expression of UEAI labeling pit cells, HK-Atpase-β labeling parietal cells (B), and AQP5 labeling antrum stem cells (C) in the antrum. Scale bar: 100 μm. Fig. S2. A graphical abstract showing the main workflow of this study. Schematic of stomach anatomy and cross-species scRNA-seq analysis in human, pig, rat and mouse. Dashed box indicates the gastric antrum epithelial region selected for scRNA-seq in all species. Fig. S3. scRNA-seq analysis reveals the landscape of epithelial cells in gastric antrum in human, pig, rat, and mouse. A The quality information of scRNA-seq from each species. B scRNA-seq analysis of gastric antral epithelium of human, pig, rat, and mouse as visualized by UMAP. C Violin plots show the range of genes identified by scRNA-seq in each species. D Expression levels of marker genes in each cell type. Pit mucous cell (GKN1, MUC5AC), basal gland mucous cell (AQP5), proliferative cell (MKI67, BIRC5, MCM6), chief cell (PGC), tuft cell (HCK, DCLK1), endocrine cell (CHGA, CHGB) and parietal cell (ATP4A, ATP4B). Color from gray to red indicates relative expression levels from low to high. Fig. S4. Distribution of F3+ cells in the pig antrum. A F3+ cells in the pig antrum glands, extending from the top to the bottom. B Expression of F3 and GSII in the pig antrum glands. Scale bar: 100 μm. C Heatmap of differentially expressed genes between BGMCs and F3+ cells. Fig. S5. The antrum epithelial cells of pig express high levels of immune response genes. A Expression of CD74 in the gastric antrum epithelial tissues of human, pig, rat, and mouse. B Heatmap shows the expression level of immune response genes in gastric antrum epithelial organoids of four species through bulk RNA-seq. C Heatmap shows the expression of immune function genes in gastric antrum epithelial organoids. D Heatmap shows the hi [file 13619_2023_171_MOESM1_ESM.pdf]

Supplemental Information

Cross-species single-cell transcriptomic analysis of animal gastric antrum reveals intense porcine mucosal immunity

Xiaodan Wang[1#], Fan Hong[2#], Haonan Li[1], Yalong Wang[2,3], Mengxian Zhang[1], Shibo Lin[4], Hui Liang[4], Hongwen Zhou[4], Yuan Liu[1] and Ye-Guang Chen[1,2, 5*]

Supplemental Figures S1-6

Supplemental Tables S1-8

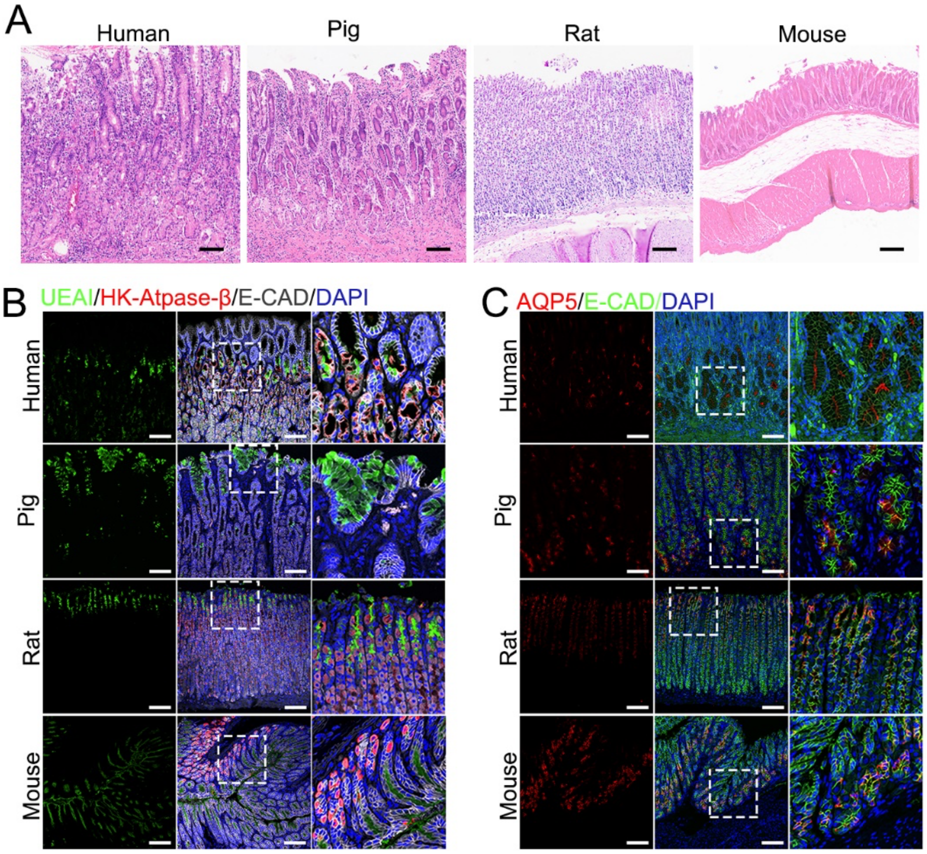

Fig. S1 Structure of the antrum of human, pig, rat, and mouse.

A H&E staining illustrating the structure of the antrum. Scale bar: 200 μm. **B, C** Expression of UEAI labeling pit cells, HK-Atpase-β labeling parietal cells (**B**), and AQP5 labeling antrum stem cells (**C**) in the antrum. Scale bar: 100 μm.

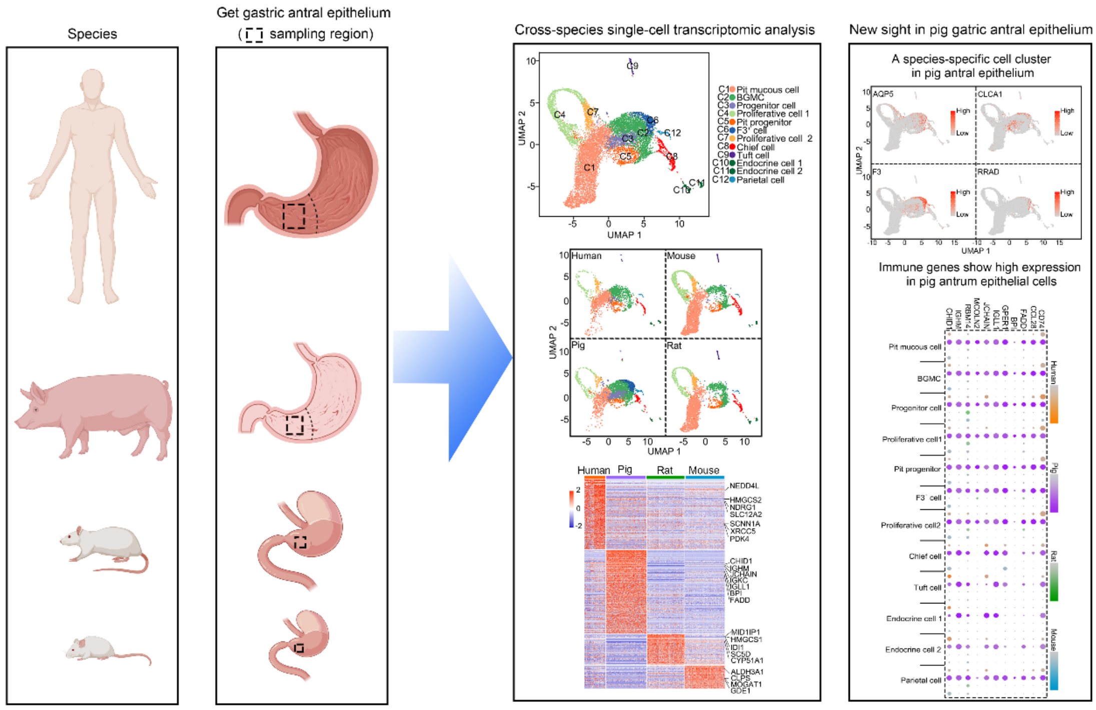

Fig. S2 A graphical abstract showing the main workflow of this study.

Schematic of stomach anatomy and cross-species scRNA-seq analysis in human, pig, rat and mouse. Dashed box indicates the gastric antrum epithelial region selected for scRNA-seq in all species.

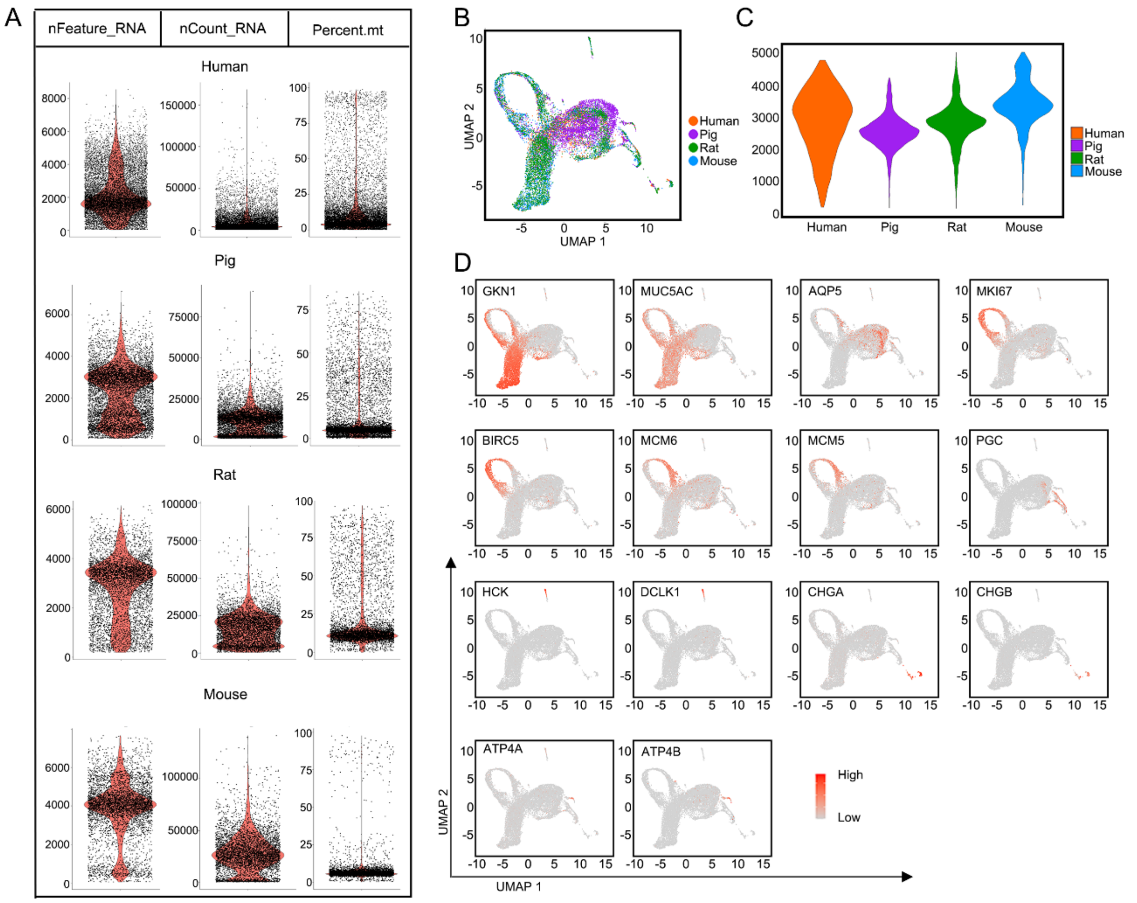

Fig. S3 scRNA-seq analysis reveals the landscape of epithelial cells in gastric antrum in human, pig, rat, and mouse.

A The quality information of scRNA-seq from each species. **B** scRNA-seq analysis of gastric antral epithelium of human, pig, rat, and mouse as visualized by UMAP. **C** Violin plots show the range of genes identified by scRNA-seq in each species. **D** Expression levels of marker genes in each cell type. Pit mucous cell (GKN1, MUC5AC), basal gland mucous cell (AQP5), proliferative cell (MKI67, BIRC5, MCM6), chief cell (PGC), tuft cell (HCK, DCLK1), endocrine cell (CHGA, CHGB) and parietal cell (ATP4A, ATP4B). Color from gray to red indicates relative expression levels from low to high.

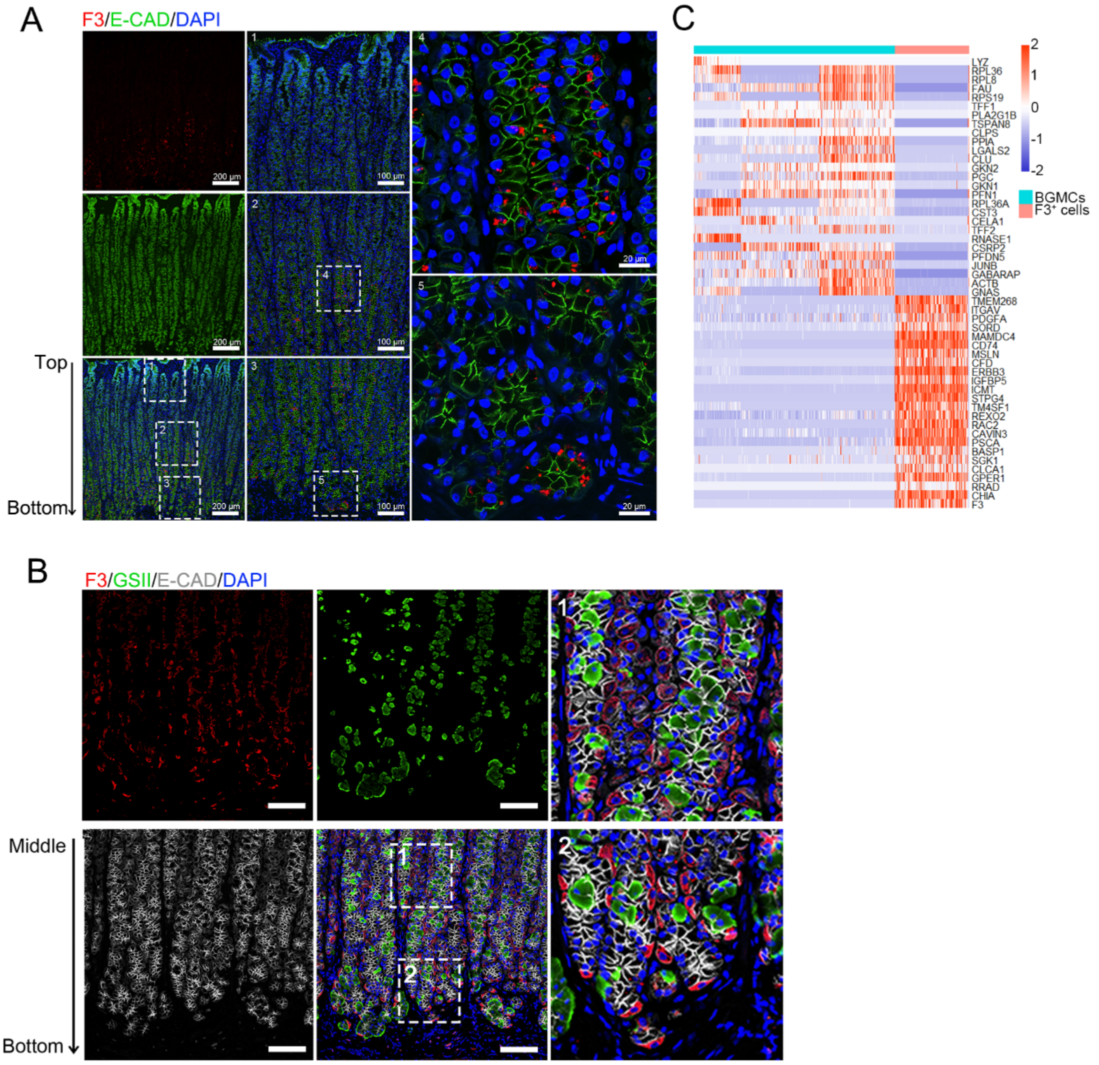

Fig. S4 Distribution of F3^{+} cells in the pig antrum.

A F3^{+} cells in the pig antrum glands, extending from the top to the bottom. **B** Expression of F3 and GSII in the pig antrum glands. Scale bar: 100 μm. **C** Heatmap of differentially expressed genes between BGMCs and F3^{+} cells.

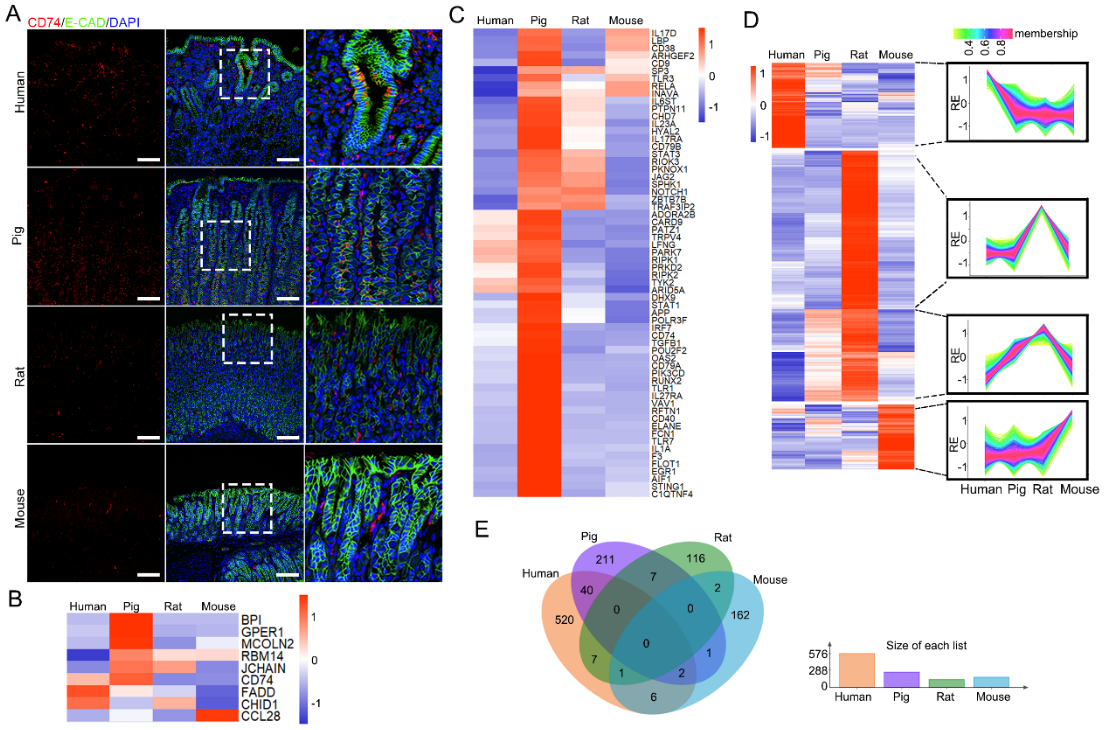

Fig. S5 The antrum epithelial cells of pig express high levels of immune response genes.

A Expression of CD74 in the gastric antrum epithelial tissues of human, pig, rat, and mouse. **B** Heatmap shows the expression level of immune response genes in gastric antrum epithelial organoids of four species through bulk RNA-seq. **C** Heatmap shows the expression of immune function genes in gastric antrum epithelial organoids. **D** Heatmap shows the highly expressed genes in organoids of human, pig, rat, and mouse (left). The change trend in each module is shown in the line chart on the right. **E** Venn diagram indicates the up-regulated gene number of organoids treated with TNFα in four species.

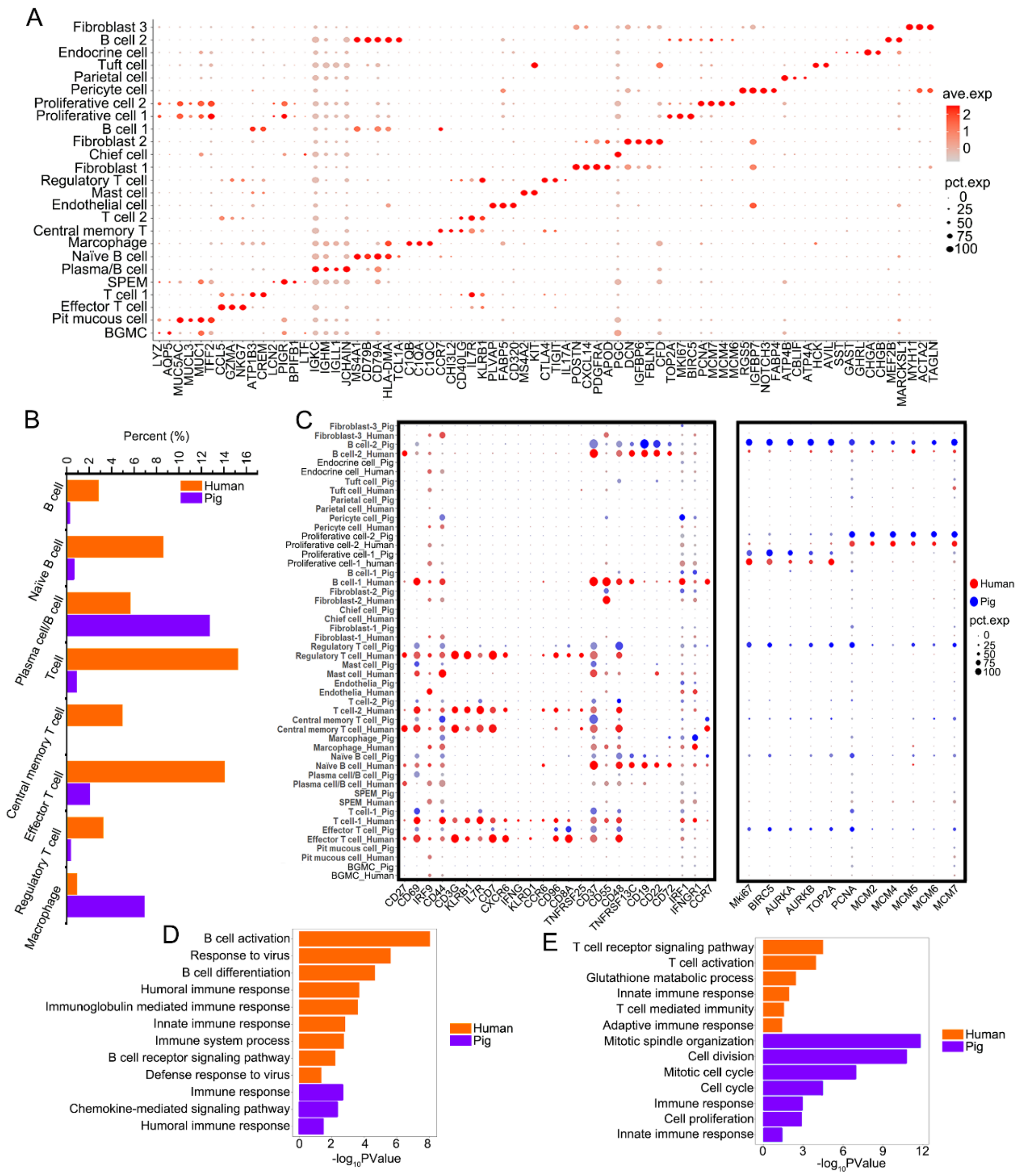

Fig. S6 T cells and B cells in pig antrum show high proliferation and low immunity compared to those in human antrum.

A Dot plot showing scaled expression level (color scale) and percent of expressing cells (point diameter) of marker genes in each cell cluster. **B** The cell ratio of immune cells (including B cells, T cells, macrophages and plasma cells) in human and pig, based on scRNA-seq. **C** Dot plot showing scaled expression level (color scale) and percent of

expressing cells (point diameter) of immunity genes (left) and proliferating genes (right) in each cell cluster of human and pig. **D, E** Function enrichment analysis of highly expressed genes in B cells (**D**) and T cells (**E**) of human and pig.

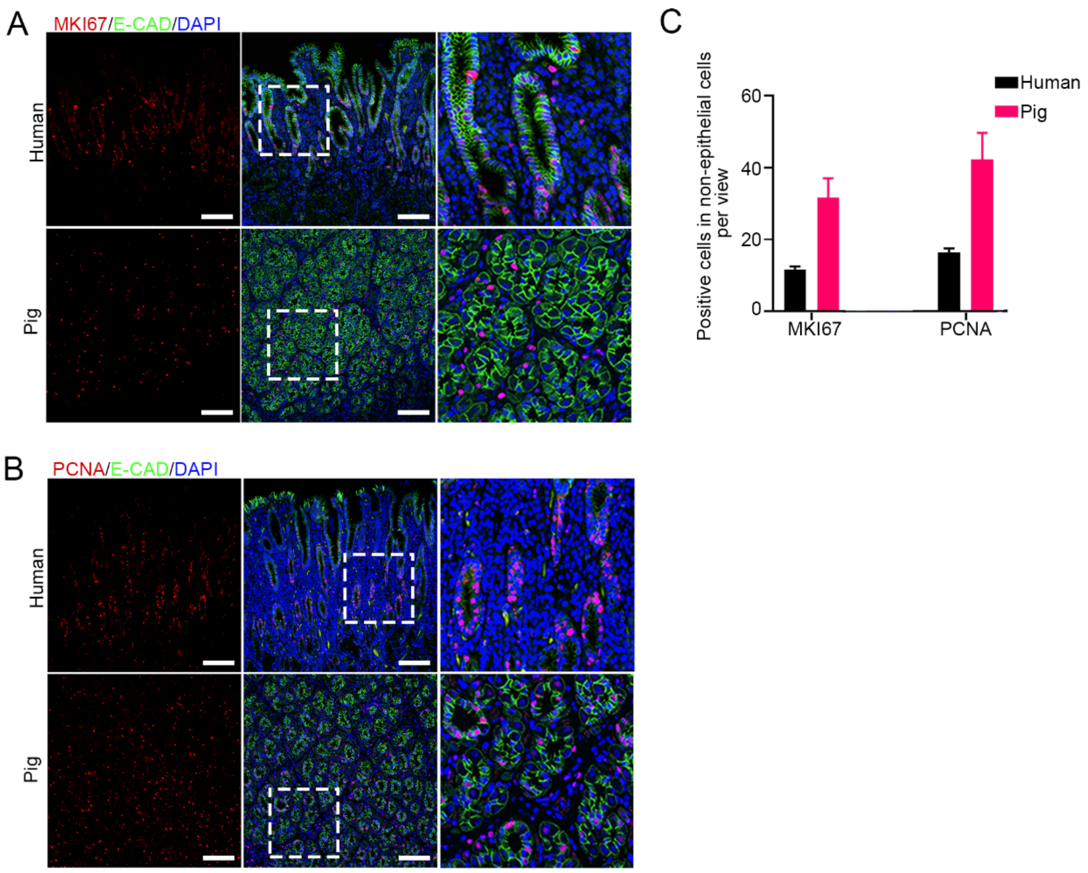

Fig. S7 Proliferative signal in antrum glands of human and pig

A Expression of MKI67 in the antrum of human and pig. **B** Expression of PCNA in the antrum of both human and pig. **C** Quantification of $MKI67^{+}$ and $PCNA^{+}$ cells in non-epithelial cells per view in panels (A) and (B). Scale bar: 100 μm.
